# No Coronary Microvascular Dysfunction After Mild COVID-19 Infection: So What Causes Long COVID Cardiac Symptoms?∗

**DOI:** 10.1016/j.jacadv.2024.100837

**Published:** 2024-02-07

**Authors:** Rebecca Kozor, Lynn Khor

**Affiliations:** a Faculty of Medicine and Health, University of Sydney, Sydney, Australia; b Department of Cardiology, Royal North Shore Hospital, St Leonards, Australia; c Department of Cardiology, Royal Prince Alfred Hospital, Camperdown, Australia

**Keywords:** cardiovascular magnetic resonance, coronary microvascular dysfunction, COVID-19

**Figure:**
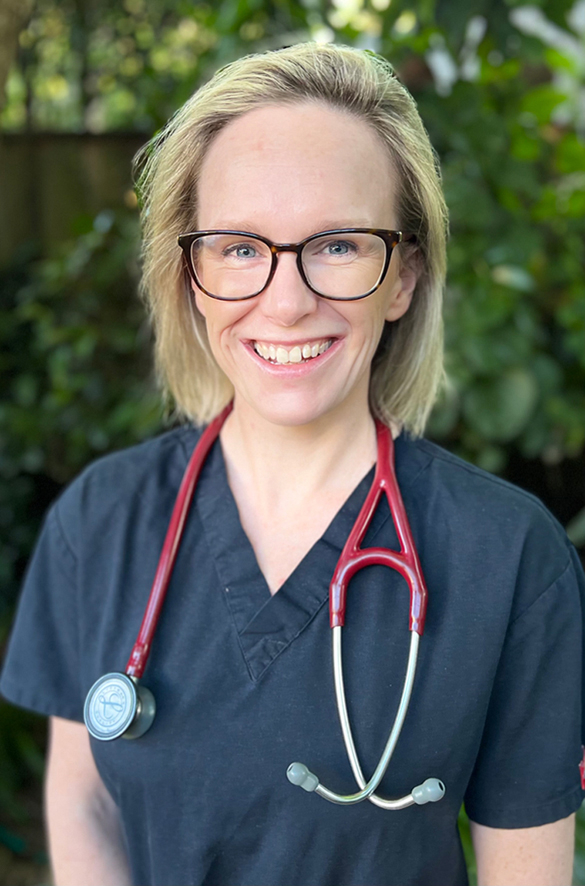

Postacute sequelae of SARS-CoV-2 infection, otherwise known as long COVID, is the continuation or development of new symptoms 3 months after the initial SARS-CoV-2 infection that last more than 2 months with no other explanation. Hospitalized SARS-CoV-2 cases are at the highest risk of developing long COVID. However, given that the vast majority of SARS-CoV-2 cases did not require hospitalization, the highest prevalence of long COVID is in nonhospitalized patients with an initial mild acute illness.^1^

Clinical manifestation of long COVID can be very variable, at times with multiorgan involvement. Several pathogenic mechanisms have been hypothesized, one of which is microvascular thrombosis and/or endothelial dysfunction.^1^ This theory is supported by a prospective, observational study showing persistent capillary rarefication and reduced vascular density 18 months after SARS-CoV-2 infection in 27 long COVID cases compared to healthy volunteers.^2^ It is notable that endothelial dysfunction is not the only cause of microvascular dysfunction; therefore, a diagnosis of microvascular dysfunction does not equate to endothelial dysfunction.

Coronary flow reserve (CFR) refers to the ratio of peak hyperemic to resting flow and is measured invasively. An abnormal CFR reflects an increase in resistance within the coronary microvasculature, which comprises the epicardial coronary arteries and coronary microvasculature. Myocardial perfusion reserve (MPR) by cardiovascular magnetic resonance (CMR) and myocardial flow reserve by positron emission tomography are thought to be the noninvasive synonyms of CFR. An abnormal CFR in the absence of epicardial stenoses is inferred to reflect coronary microvascular dysfunction (CMD). Similarly, a reduction of myocardial blood flow at stress (sMBF) or MPR in the absence of obstructive coronary artery disease implies CMD.

It is generally accepted that quantitative rather than qualitative CMR perfusion mapping is necessary to detect CMD. There are several different acquisition methods and postprocessing methods for quantitative perfusion. To date, there is a lack of consensus on optimal acquisition and postprocessing techniques for sMBF and MPR.

In this issue of *JACC: Advances*, Karagodin et al^3^ report no significant differences in myocardial blood flow at rest or sMBF nor MPR using CMR between patients with prior mild SARS-CoV-2 infection (n = 30) and matched controls (n = 26). They subsequently infer that microvascular function after a mild SARS-CoV-2 infection is comparable to that without a prior infection.

This study used a 1.5-T scanner (SIGNA Artist, GE Healthcare) and intravenous Regadenoson as the pharmacological stress agent. CMR perfusion imaging was performed using a fully quantitative perfusion sequence (dual sequence dual-bolus technique with computer-based postprocessing using Fermi deconvolution to create pixel-wise myocardial blood flow quantification), as previously published and validated in other cohorts. It took patients with a prior mild SARS-CoV-2 infection and prospectively scanned them at a median time interval of 5.6 months (ie, quite remote from their infection). Of these, 57% had cardiac symptoms (mainly chest pain and dyspnea), which were significantly more frequent than the control group (*P* < 0.001). Additionally, although 30% (9/30) of patients had at least one myocardial abnormality detected on CMR (eg, evidence of scar, increased T1, increased T2), this was similar to the control group. Therefore, there were no CMR features to explain the increased cardiac symptoms post-mild SARS-CoV-2 infection.

As highlighted by the authors, the characteristics of studies looking at the association between CMD and cardiac symptoms post-SARS-CoV-2 infection are heterogeneous. These differing factors include median time interval from SARS-CoV-2 infection to myocardial perfusion imaging, imaging modality, CMR scanner and perfusion imaging protocol, and severity of initial SARS-CoV-2 infection. These differences make it difficult for direct comparison or pooled analyses to investigate the potential role of CMD post-SARS-CoV-2.

This study suggests that patients with prior mild SARS-CoV-2 infection do not have CMD; however, sMBF does not reflect systemic microvascular and endothelial dysfunction. A different view would be that sMBF and MPR may not be the best modalities to detect microvascular dysfunction in this specific population. From a pathophysiological perspective, manifestation of endothelial dysfunction may not equate to reduced sMBF. Perhaps a different test for coronary endothelial dysfunction would be an acetylcholine challenge, which could be simulated by cold pressor testing. Given that chest pain is the more common clinical manifestation of CMD, it would have been interesting to examine if sMBF was reduced specifically in the 30% of cases where chest pain was reported (although the numbers were small in this subgroup). This is where future studies should focus.

Considering the abovementioned limitations, this study provides further insights into prior mild SARS-CoV-2 by showing no abnormalities in myocardial perfusion compared to matched controls, despite the high frequency of cardiac symptoms. The question still remains: what causes long COVID cardiac symptoms?

## Funding support and author disclosures

The authors have reported that they have no relationships relevant to the contents of this paper to disclose.
